# Educational attainment and mortality in schizophrenia

**DOI:** 10.1111/acps.13407

**Published:** 2022-02-18

**Authors:** Martin Tesli, Eirik Degerud, Oleguer Plana‐Ripoll, Kristin Gustavson, Fartein Ask Torvik, Eivind Ystrom, Helga Ask, Natalia Tesli, Anne Høye, Camilla Stoltenberg, Ted Reichborn‐Kjennerud, Ragnar Nesvåg, Øyvind Næss

**Affiliations:** ^1^ Norwegian Institute of Public Health Oslo Norway; ^2^ Norwegian Centre for Mental Disorders Research Oslo University Hospital Oslo Norway; ^3^ National Institute of Occupational Health Oslo Norway; ^4^ Department of Economics and Business Economics National Centre for Register‐Based Research Aarhus University Aarhus V Denmark; ^5^ Department of Clinical Epidemiology Aarhus University and Aarhus University Hospital Aarhus N Denmark; ^6^ Department of Psychology University of Oslo Oslo Norway; ^7^ PharmacoEpidemiology and Drug Safety Research Group School of Pharmacy University of Oslo Oslo Norway; ^8^ Division of Mental Health and Substance Abuse University Hospital of North Norway Tromsø Norway; ^9^ Department of Clinical Medicine The Arctic University of Norway Tromsø Norway; ^10^ Department of Global Public Health and Primary Care University of Bergen Bergen Norway; ^11^ Institute of Clinical Medicine University of Oslo Oslo Norway; ^12^ Institute of Health and Society University of Oslo Oslo Norway

**Keywords:** cardiovascular disease, education, mortality, schizophrenia, socioeconomic

## Abstract

**Background:**

Individuals suffering from schizophrenia have a reduced life expectancy with cardiovascular disease (CVD) as a major contributor. Low educational attainment is associated with schizophrenia, as well as with all‐cause and CVD mortality. However, it is unknown to what extent low educational attainment can explain the increased mortality in individuals with schizophrenia.

**Aim:**

Here, we quantify associations between educational attainment and all‐cause and CVD mortality in individuals with schizophrenia, and compare them with the corresponding associations in the general population.

**Method:**

All Norwegian citizens born between January 1, 1925, and December 31, 1959, were followed up from January 1, 1990, to December 31, 2014. The total sample included 1,852,113 individuals, of which 6548 were registered with schizophrenia. We estimated hazard ratios (HR) for all‐cause and CVD mortality with Cox models, in addition to *life years lost*. Educational attainment for index persons and their parents were included in the models.

**Results:**

In the general population individuals with low educational attainment had higher risk of all‐cause (HR: 1.48 [95% CI: 1.47–1.49]) and CVD (HR: 1.59 [95% CI: 1.57–1.61]) mortality. In individuals with schizophrenia these estimates were substantially lower (all‐cause: HR: 1.13 [95% CI: 1.05–1.21] and CVD: HR: 1.12 [95% CI: 0.98–1.27]). Low educational attainment accounted for 3.28 (3.21–3.35) life years lost in males and 2.48 (2.42–2.55) years in females in the general population, but was not significantly associated with life years lost in individuals with schizophrenia. Results were similar for parental educational attainment.

**Conclusions:**

Our results indicate that while individuals with schizophrenia in general have lower educational attainment and higher mortality rates compared with the general population, the association between educational attainment and mortality is smaller in schizophrenia subjects than in the general population.

**Significant outcomes**

- Individuals with schizophrenia have a reduced life expectancy and lower educational attainment than the general population.
- We found a smaller association between educational attainment and mortality in individuals with schizophrenia than the general population.
- Parents' educational attainment is similar between individuals with schizophrenia and the general population, supporting the hypothesis of social drift.

**Limitations**

- These findings might have limited generalizability to other countries, in particular those with larger socioeconomic differences than Norway.
- The relationship between educational attainment and mortality might differ between schizophrenia and other mental disorders

## 1 INTRODUCTION

Individuals with schizophrenia not only suffer from disturbing and often disabling symptoms such as hallucinations, delusions, and thought distortions but also live approximately 10 years shorter than the general population.^1^, ^2^ While mortality rates from unnatural causes such as accidents and suicide have been reduced in schizophrenia during the last decades, this does not hold true for natural causes, among which cardiovascular disease (CVD) has been shown to be a major contributor.^1^, ^3^ In the general population, CVD mortality has been halved in most industrialized countries since the late 1970s.^4^ However, individuals with schizophrenia have not benefited from improvements in CVD prevention and health care to the same degree as the rest of the population.^3^ One recent report found that excess life years lost due to CVD had increased in schizophrenia compared with the general population from 1995 to 2015.^1^

In the general population, despite a decline in premature mortality and CVD,^4^ social inequalities in health have remained high in high‐income countries with some improvements in recent years.^5^ Most of the observed population level decline and social inequality in CVD are related to general improvement and social distribution of established CVD risk factors.^5^, ^6^

Putative mechanisms for premature CVD death in individuals with schizophrenia include patient‐related factors such as adverse effects of antipsychotic medication,^7^ common genetic risk,^8^ and an unhealthy lifestyle,^9^ as well as system‐related factors such as under‐diagnostics and under‐treatment of somatic conditions, like previously reported from other countries^10^ and from Norway by our group.^11^, ^12^ Further, the recognition of CVD risk factors in patients with psychotic disorders by healthcare providers might in general occur too seldom and late.^10^ Both patient‐related and system‐related factors could be indexed by a measure of socioeconomic position (SEP) such as educational attainment. Disadvantaged SEP has consistently been associated with increased risk of schizophrenia,^13^ but it is uncertain whether socioeconomic factors are causally involved in the development of schizophrenia in early life (social causation hypothesis), whether reduced social function leading to lower SEP in adulthood is caused by schizophrenia symptoms (social drift hypothesis),^13^, ^14^ or whether both these hypotheses are true. Although lower academic achievement and premorbid cognitive dysfunction have been reported in schizophrenia patients,^15^ this could be explained by social causation as well as social drift due to premorbid subthreshold symptoms. Further, parents of individuals with schizophrenia have been found to have similar educational attainment as parents of individuals without schizophrenia, indicating social drift as a possible mechanism.^13^

The impact of SEP on the mortality gap in schizophrenia remains, to the best of our knowledge, unknown. Better understanding of the extent to which socioeconomic factors explain the increased mortality in schizophrenia patients could guide researchers in further exploration on the underlying mechanisms, which, in turn, may provide stronger evidence for clinicians and health policymakers when designing intervention plans and programs.

In the current study, we estimated the associations between educational attainment and all‐cause and CVD‐related mortality in individuals with schizophrenia in a unique data set comprising the entire Norwegian population. The study extends our previous findings of an educational gradient for CVD risk factors^16^ and CVD‐related mortality^17^ in the general population. Moreover, educational attainment as a measure of SEP is relatively easy to interpret and translate into other countries and cultures, in contrast to, for example, living conditions and income distribution.

To address the potential reverse causality between schizophrenia and educational length, we included the educational attainment of index persons as well as their parents in the analyses.

The associations between education/parental education and mortality in individuals with schizophrenia were compared with the corresponding associations in the general population. Additionally, we estimated *life years* lost associated with low educational attainment in individuals with schizophrenia and in the general population.^18^ By comparing the educational attainment and parental educational attainment of individuals with and without schizophrenia, we also investigated the “social causation” vs. “social drift” hypotheses.

As educational attainment per se does probably not capture the entire socioeconomic variance relevant for the current research question, as a sensitivity analysis, we included a composite measure of SEP indicators through the life course in place of education in the analyses described above. This cumulative life course SEP measure, for which we have recently demonstrated a fine‐graded association with CVD‐related mortality rates in the Norwegian population,^19^ comprises household conditions from three decades, educational attainment and household income.^19^, ^20^

Due to paucity of research on the relation between educational attainment, mortality, and schizophrenia, our initial hypothesis was that educational attainment plays an equally large role for mortality rates in individuals with schizophrenia as in the general population.

### 1.1 Aims of the study

The main aim of the current study was to determine to what extent low educational attainment can explain the increased mortality in individuals with schizophrenia. We investigated this research question by quantifying the associations between educational attainment and mortality in individuals with schizophrenia and comparing them with the corresponding associations in the general population. As a second aim we wanted to investigate the “social causation” vs. “social drift” hypotheses by comparing the educational and parental educational attainment in individuals with vs. without a diagnosis of schizophrenia.

## 2 MATERIAL AND METHODS

### 2.1 Study population

All Norwegian citizens born between January 1, 1925, and December 31, 1959, were followed up prospectively from January 1, 1990, until emigration, death from any cause, or December 31, 2014.

Thus, the age range at study start was 30–65 years. The total sample included 1,852,113 individuals, of which 6548 were registered with schizophrenia. For further information on inclusion, exclusion and missingness, please see Figure S1.

### 2.2 Data linkage and ethical approval

We linked data from the National Population Registry, the National Insurance Scheme (NIS), the National Educational Database and the Cause of Death Registry using personal identification numbers (PINs) and a trusted third party (Statistics Norway). The data linkage and the research project were approved by the Regional Ethics Committee South‐East (11/1676).

### 2.3 Diagnostic assessment of schizophrenia

All residents of Norway have been insured by the NIS since 1967. Some earlier types of social benefits were continued through NIS from this year. The NIS provides disability benefits for people with medical conditions of sufficient severity to be an economic burden. This includes a basic benefit for substantial expenses as a result of the disability, an attendance benefit (financial compensation for use of services or nursing), and a disability pension for persons above 18 years whose working capacity is permanently reduced by at least 50%. The benefits provided are independent of previous income or wealth. The diagnoses associated with the disability are based on medical examinations by physicians and classified according to the International Classification of Diseases (ICD). In the current study, diagnostic assessment was based on disability pensions from NIS according to schizophrenia criteria in ICD‐8/9 (295.0–295.9) for the period 1983–1998 and ICD‐10 (F20.0‐F20.9) from 1998 onwards. NIS has previously been shown to be a valid data source for identification of a range of neuropsychiatric disorders,^21^, ^22^ and we have previously used schizophrenia diagnoses from NIS for the purpose of investigating the relationship between intrauterine growth restriction and schizophrenia.^21^

### 2.4 Educational attainment

We obtained information on the highest level of attained education ever recorded until 2011 (National Educational Database) for the index persons as well as their parents. The Norwegian education system is comparable to those of most Western countries and higher education follows the standards of the Bologna system. Moreover, the Norwegian education system has been universal, mandatory and free of charge since the 19th century, hence including all the participants in this study. Information from the National Educational Database is available at individual level from 1970 onwards. A person's highest attained educational level was classified as either basic (up to 7 years), representing compulsory primary school or lower levels, or higher – representing completion of secondary school, or a college or university degree. These categories correspond to the international standard classification of education (ISCED) 2011 categories 0–1 vs. 2–8. The rationale for dichotomizing educational attainment was to maintain statistical power, particularly in the schizophrenia group, as well as increasing clarity and interpretability of the results from the Cox regressions, Kaplan‐Meier survival curves and life years lost analyses. A more fine‐grained educational ladder (0–8) is presented in Table 1. We also ran a set of additional analyses, in which educational attainment was treated both as a Likert scale (0–8) and divided in 3 categories (low: 0–1, middle: 2, high: 3–8 according to ISCED 2011 categories). For the dichotomization as well as the tripartition of educational attainment, we sought to obtain a similar number of participants in each group.

**TABLE 1:** Demographics

Variable	Schizophrenia	Remaining population	*p* Value	Test statistic
Sex females	2682 (41.0%)	905,681 (49.1%)	<10^−16^	x^2^ = 171.6
Sex males	3866 (59.0%)	939,884 (50.9%)	<10^−16^	x^2^ = 171.6
Age at study start (mean, SD)	45.6 (10.2)	45.2 (10.0)	0.001	t = −3.3
*N* total deaths (1990–2015)	3143 (48.0%)	384,502 (20.8%)	<10^−16^	x^2^ = 2907.9
*N* CVD deaths (1990–2015)	988 (15.1%)	113,679 (6.2%)	<10^−16^	x^2^ = 894.2
Education until 2011 (1/0)	44.7%	30.9%	<10^−16^	x^2^ = 573.9
Education until 2011 (1–8) (mean, SD)	3.07 (1.6)	3.62 (1.4)	<10^−16^	t = 32.0
Educational level				
1 (Preprimary education)	98 (1.5%)	14,105 (0.8%)	—	—
2 (Primary education)	2781 (43.2%)	539,045 (30.1%)	—	—
3 (Lower secondary education)	1931 (30.0%)	519,810 (29.0%)	—	—
4 (Higher secondary education)	764 (11.9%)	276,076 (15.4%)	—	—
5 (Postsecondary education)	95 (1.5%)	44,995 (2.5%)	—	—
6 (Bachelor's or equivalent)	64 (10.0%)	295,438 (16.5%)	—	—
7 (Master's or equivalent)	120 (1.9%)	91,349 (5.1%)	—	—
8 (Doctoral or equivalent)	9 (0.1%)	11,040 (0.6%)	—	—
Parental education (1/0)	49.3%	48.7%	0.36	x^2^ = 0.9
Parental education (1–8) (mean, SD)	3.01 (1.4)	2.95 (1.3)	0.002	t = −3.15
1 (Preprimary education)	6 (0.1%)	1165 (0.1%)	—	—
2 (Primary education)	2715 (49.2%)	727,611 (48.6%)	—	—
3 (Lower secondary education)	1636 (29.6%)	472,415 (31.5%)	—	—
4 (Higher secondary education)	463 (8.4%)	136,120 (9.1%)	—	—
5 (Postsecondary education)	59 (1.1%)	16,988 (1.1%)	—	—
6 (Bachelor's or equivalent)	390 (7.1%)	94,810 (6.3%)	—	—
7 (Master's or equivalent)	239 (4.3%)	46,450 (3.1%)	—	—
8 (Doctoral or equivalent)	11 (0.2%)	1821 (0.1%)	—	—
SEP measures (1 = high risk, 0 = low risk)				
Census 1960				
Water closet (1/0)	58.8%	56.6%	5 × 10^−4^	x^2^ = 12.1
Bathroom (1/0)	53.5%	50.5%	3 × 10^−6^	x^2^ = 21.9
Telephone (1/0)	62.2%	61.0%	0.04	x^2^ =4.4
Ownership status (1/0)	33.6%	35.9%	3 × 10^−4^	x^2^ =13.2
Rooms per household capita (1/0)	37.2%	36.8%	0.6	x^2^ = 0.25
Type of dwelling (1/0)	18.4%	16.9%	9 × 10^−5^	x^2^ = 15.4
Census 1970				
Water closet (1/0)	30.0%	22.4%	<10^−16^	x^2^ = 201.7
Bathroom (1/0)	32.3%	24.2%	<10^−16^	x^2^ = 210.6
Telephone (1/0)	52.4%	51.5%	0.1	x^2^ = 2.1
Ownership status (1/0)	40.6%	41.9%	0.05	x^2^ = 3.9
Rooms per household capita (1/0)	61.0%	56.8%	4 × 10^−11^	x^2^ = 43.4
Type of dwelling (1/0)	20.9%	17.2%	1 × 10^−14^	x^2^ = 59.8
Census 1980				
Water closet (1/0)	15.7%	7.0%	<10^−16^	x^2^ = 617.5
Bathroom (1/0)	15.3%	6.3%	<10^−16^	x^2^ = 730.4
Telephone (1/0)	41.0%	34.5%	<10^−16^	x^2^ = 98.0
Ownership status (1/0)	26.3%	21.2%	1 × 10^−15^	x^2^ = 64.3
Rooms per household capita (1/0)	83.5%	80.0%	2 × 10^−11^	x^2^ = 45.1
Type of dwelling (1/0)	25.8%	16.4%	<10^−16^	x^2^ = 337.4
Household income 1990 (1/0)	70.0%	24.9%	<10^−16^	x^2^ = 6747.2
SEP 0–20 (mean, SD)	8.01 (3.02)	6.94 (2.62)	<10^−16^	t = −22.4
High SEP [0–9]	2762 (69.3%)	1,053,632 (83.7%)	<10^−16^	x^2^ = 627.0
Low SEP [10–20]	1221 (30.7%)	205,034 (16.3%)	<10^−16^	x^2^ = 627.0

Note

All Norwegian citizens born between January 1, 1925, and December 31, 1959, were followed up from January 1, 1990, to December 31, 2014, (total *N* = 1,852,113, *N* individuals with schizophrenia = 6548). Education (1/0): 1 = none/primary school; 0 = secondary school or higher.

Abbreviations: CVD, cardiovascular disease; SD, standard deviation; SEP, socioeconomic position.

### 2.5 Life course SEP

We obtained a cumulative measure of life course SEP for each citizen by combining indicators on household conditions (i.e., type of dwelling, apartment block, row or detached house, rooms per household capita, ownership status, telephone ownership, access to water closet and bath inside the dwelling) from mandatory population and household censuses in 1960, 1970, and 1980, household income from the census in 1990 and the highest level of obtained education ever recorded until 2011 (National Educational Database). The household conditions, household income, and education level provided a total of 20 indicators, which were scored (0 or 1) and given equal weight by summing the scores to construct the cumulative index (range 0–20). A high score indicated disadvantage and low life course SEP, as described in more detail in previous studies.^19^, ^20^ Here, we treated SEP both as a continuous and as a binary variable, dichotomized into low SEP [10–20] and high SEP [0–9]. For the sake of interpretative clarity and statistical power, we conducted the survival analyses (described below) mainly with a dichotomous SEP measure. For further details, please see Table 1 and Figure S1.

### 2.6 Cause of death

The Norwegian Cause of Death Registry, which contains information on causes of death since 1951, provided outcome data using ICD‐9 and ICD‐10. Here we included all‐cause and CVD mortality (1990–1995: ICD‐9: 390–459; 1996–2014: ICD‐10: I00‐I99). The registry is almost exclusively based on certificates filled out on‐site by medical doctors, and in the few cases in which autopsies are performed, 32% of deaths are reclassified over major ICD‐10 chapters.^23^

### 2.7 Statistical analyses

#### 2.7.1 Demographics

In the demographic analyses we described the study population (sex, age, death, educational attainment, parental educational attainment and other SEP measures) according to being diagnosed with schizophrenia or not. Continuous variables were presented as mean with standard deviation (SD) and categorical variables as counts (%). *T* tests and chi‐squared tests assessed differences between the groups.

### 2.8 Main analyses

#### 2.8.1 Cox regressions

In the survival analyses in the total sample, we estimated hazard ratios (HRs) with 95% confidence intervals (CIs) using Cox proportional hazard models. Based on visual inspection of plots of Schoenfeld residuals against time, there was no indication of deviation from the proportional hazard assumption. Since age of onset for schizophrenia was not available from the NIS data source, schizophrenia was treated as a time‐fixed variable in the analyses. Schizophrenia, educational/parental educational attainment (low vs. high), male sex and age at start of follow‐up were used as predictors in the Cox regression model and all‐cause/CVD‐related death were outcome variables. Kaplan‐Meier survival curves starting at age 30 years were produced for all these analyses. An interaction term was added to the Cox models to assess potential differences between the associations between educational/parental educational attainment and mortality in individuals with and without schizophrenia.

Hazard ratios for educational/parental educational attainment was also estimated within the schizophrenia group in a model adjusted for sex and age at start of follow‐up.

Additionally, we performed Cox regression analyses with education/parental education as a continuous scale from 0 to 8 for CVD as well as all‐cause mortality, followed by interaction analyses. We also conducted stratified Cox regression analyses in each of the 3 education/parental education categories.

### 2.9 Life years lost analyses

We applied the *life years lost* method^2^ to estimate excess life years lost at age 30 years due to all‐cause and CVD mortality for 1. individuals with schizophrenia (exposed) compared with individuals without schizophrenia (unexposed), 2. individuals without schizophrenia with low educational/parental educational attainment (exposed) compared with individuals without schizophrenia with high educational/parental educational attainment (unexposed), 3. individuals with schizophrenia and low educational/parental educational attainment (exposed) compared with individuals with schizophrenia and high educational/parental educational attainment (unexposed). All analyses estimating life years lost were performed in males and females separately. We also conducted life years lost analyses where we compared the highest third with the lowest third of the 3 education/parental education categories.

### 2.10 Sensitivity analyses

As sensitivity analyses, we performed Cox regressions and life years lost analyses for the dichotomized life course SEP measure in place of educational attainment.

Cox regressions and life years lost analyses were conducted in *R* statistical software using *R studio* (https://rstudio.com/) with additional use of the packages *survival* (https://cran.r‐project.org/web/packages/survival/), *survminer* (https://cran.r‐project.org/web/packages/survminer/) and *lillies* (https://cran.r‐project.org/web/packages/lillies/).^2^, ^18^

## 3 RESULTS

### 3.1 Demographics

A larger proportion of individuals with schizophrenia were male compared with the remaining population (59.0% vs. 50.9%). Mean age at start of follow‐up (1990) was similar in individuals with schizophrenia (45.6 years, SD 10.2) as in those without schizophrenia (45.2 years, SD 10.0). The percentage of all‐cause and CVD deaths from 1990 to 2015 were higher in individuals with schizophrenia (all‐cause 48.0%, CVD deaths 15.1%) than in those without schizophrenia (all‐cause 20.8%, CVD deaths 6.2%). Education on the scale from 1 to 8 as well as dichotomized were lower in the schizophrenia group than in the remaining population (44.7% vs. 30.9% with primary school or less). However, parental education on the scale from 1 to 8 was marginally higher in individuals with schizophrenia than in the general population, and similar in the two groups when dichotomized (49.3% vs. 48.7% with primary school or less). See Table 1 for further details.

### 3.2 Main analyses

#### 3.2.1 Cox regressions

**FIGURE 1:**
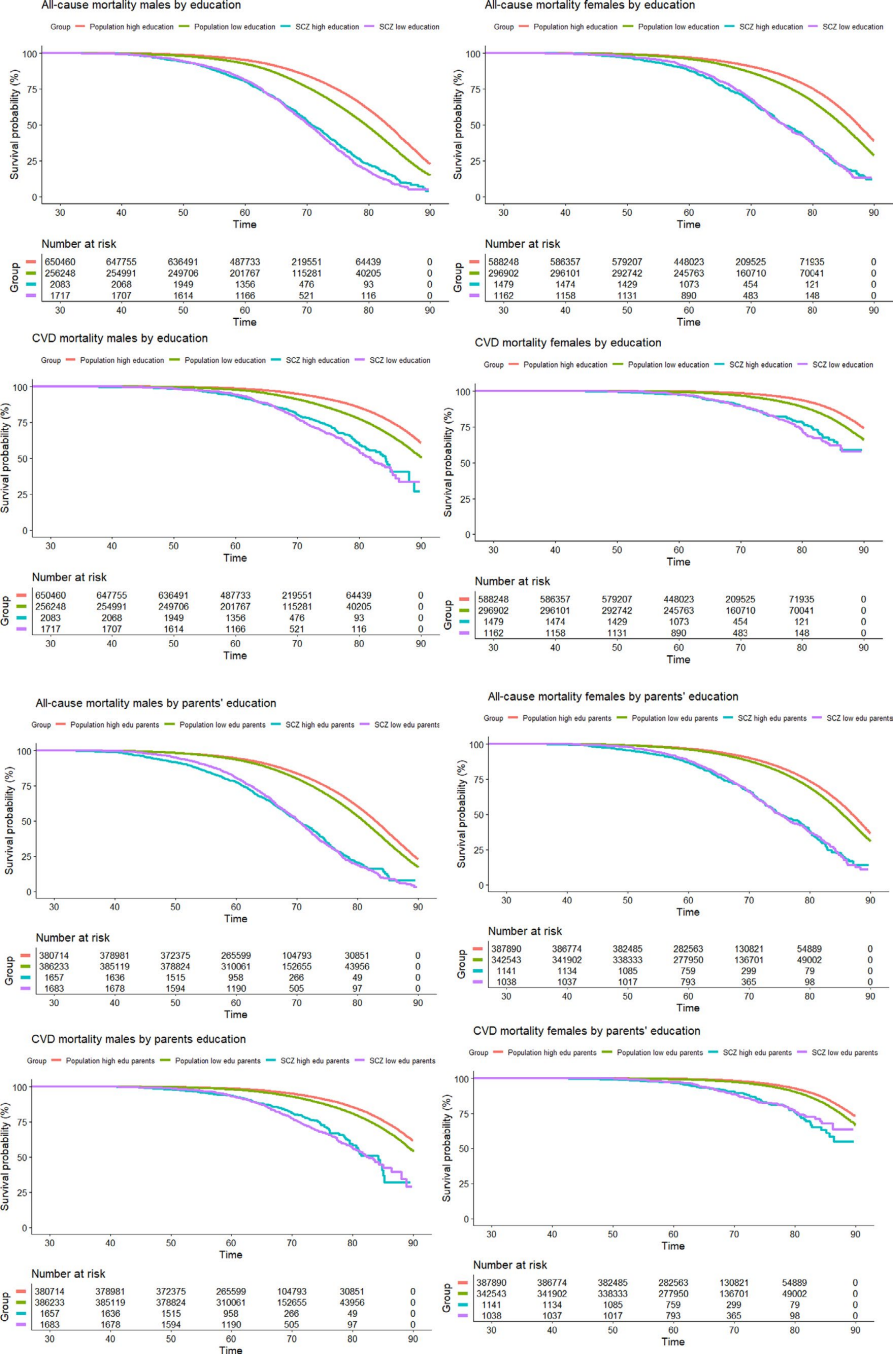
Kaplan–Meier curves. Survival probability (*y* axis) for all‐cause and cardiovascular disease (CVD) death, divided by sex, from the Norwegian population (Total *N* = 1,852,113, *N* schizophrenia patients = 6548). Survival time *x* axis = years. Abbreviations: Edu, educational attainment; Low education, primary school or less; SCZ, schizophrenia

In a Cox regression model including schizophrenia, sex, educational attainment and age at start of follow‐up, individuals with schizophrenia were at a higher risk of all‐cause (HR = 2.89 [95% CI: 2.89–3.00]) and CVD mortality (HR = 3.04 [95% CI: 2.85–3.24]) compared with those without schizophrenia. Individuals in the low education group were at a higher risk of all‐cause (HR = 1.48 [95% CI: 1.47–1.49]) and CVD mortality (HR = 1.59 [95% CI: 1.57–1.61]) compared with the high education group. When adding an interaction term to the Cox model, there were significant interaction effects between schizophrenia and low educational attainment for all‐cause (HR = 0.71 [95% CI: 0.66–0.76]) as well as CVD mortality (HR = 0.64 [95% CI: 0.56–0.72]), that is, the associations between low educational attainment and all‐cause and CVD mortality were stronger in individuals without schizophrenia than in individuals with schizophrenia. Individuals with schizophrenia and low educational attainment were at marginally increased risk of all‐cause death (HR = 1.13 [95% CI: 1.05–1.21]) and CVD death (HR = 1.12 [95% CI: 0.98–1.27]) compared with individuals with schizophrenia and high educational attainment. We found similar results when using parental educational attainment in place of educational attainment of the index person in the analyses described above. Main HR estimates for educational attainment with 95% CI are presented in Table 2 and for parental educational attainment in Table 3. The survival probabilities for CVD and all‐cause death are presented in Kaplan‐Meier curves in Figure 1.

**TABLE 2:** Cox regressions mutually adjusted for schizophrenia, sex, education (dichotomized into high and low) and age at start for follow‐up in the Norwegian population aged 30–65 January 1, 1990, with follow‐up to December 31, 2014, (total *N* = 1,852,113, *N* individuals with schizophrenia = 6548)

Sample	Predictor variable	CVD mortality HR (95% CI)	All‐cause mortality HR (95% CI)
Total cohort (*N* = 1,852,113)	Schizophrenia	3.04 (2.85–3.24)	2.89 (2.79– 3.00)
	Male sex	2.28 (2.25–2.30)	1.71 (1.70–1.72)
	Low education	1.59 (1.57–1.61)	1.48 (1.47–1.49)
	Schizophrenia × low education	0.64 (0.56–0.72)	0.71 (0.66–0.76)
Schizophrenia patients (*N* = 6548)	Male sex	2.03 (1.77–2.33)	1.61 (1.49–1.73)
	Low education	1.12 (0.98–1.27)	1.13 (1.05–1.21)

Note

Left: CVD (cardiovascular disease) related mortality. Right: All‐cause mortality.

**TABLE 3:** Cox regressions mutually adjusted for schizophrenia, sex, parents' highest education (dichotomized into high and low) and age at start for follow‐up in the Norwegian population aged 30–65 January 1, 1990, with follow‐up to December 31, 2014, (total *N* = 1,852,113, *N* individuals with schizophrenia = 6548).

Sample	Predictor variable	CVD mortality HR (95% CI)	All‐cause mortality HR (95% CI)
Total cohort (*N* = 1,852,113)	Schizophrenia	3.35 (3.12–3.56)	3.14 (3.02– 3.27)
	Male sex	2.15 (2.12–2.18)	1.63 (1.62–1.64)
	Low parent education	1.33 (1.32–1.35)	1.23 (1.22–1.24)
	Schizophrenia × low parent education	0.72 (0.62–0.83)	0.79 (0.73–0.85)
Schizophrenia patients (*N* = 6548)	Male sex	2.07 (1.77–2.42)	1.62 (1.49–1.76)
	Low parent education	1.08 (0.94–1.25)	1.08 (1.00–1.17)

Note

Left: CVD (cardiovascular disease) related mortality. Right: All‐cause mortality.

When conducting Cox regressions with education as a Likert scale from 0 to 8, we found similar results, both for education and parental education in CVD and all‐cause mortality (Tables S1 and S2). As for the analyses including 3 categories of educational/parental education, we found the same pattern of stronger association between schizophrenia and all‐cause/CVD mortality in those with higher educational and parental educational attainment (Tables S3 and S4).

#### 3.2.2 Life years lost analyses

**FIGURE 2:**
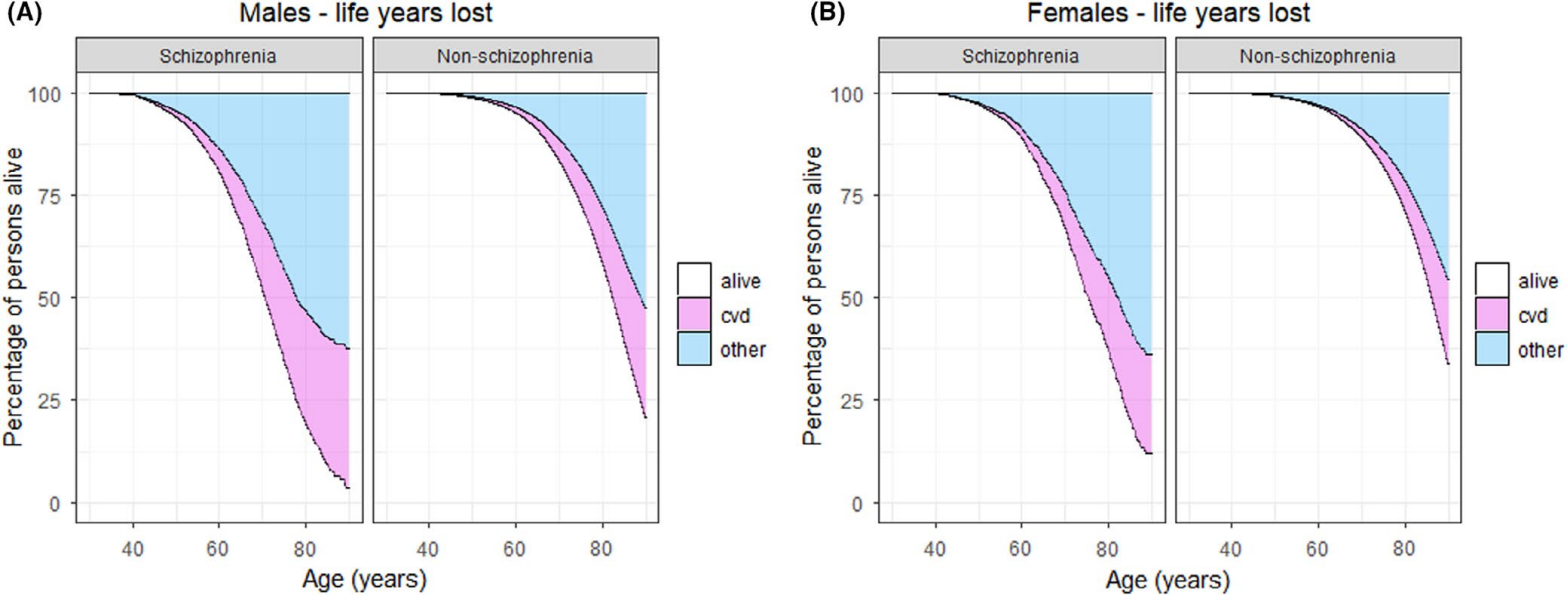
Excess life years lost for the Norwegian population aged 30–65 on January 1, 1990, with follow‐up to December 31, 2014 (total *N* = 1,852,113, *N* individuals with schizophrenia = 6548). Survival time *x* axis = years. CVD = life years lost due to cardiovascular related disease. Other = life years lost due all other causes of death. (A) Males with schizophrenia compared with males without schizophrenia. (B) Females with schizophrenia compared with females without schizophrenia

Compared with males without schizophrenia, life years lost at age 30 years were 9.96 (95% CI: 9.55–10.37) for males with schizophrenia, of which 3.21 (95% CI: 2.82–3.65) years could be attributed to CVD. For females with schizophrenia 8.59 (95% CI: 8.08–9.10) years were lost compared with females without schizophrenia, of which 2.32 (95% CI: 1.91–2.77) years could be attributed to CVD (Figure 2). Males without schizophrenia with low educational attainment lost 3.28 (95% CI: 3.21–3.35) years compared with males without schizophrenia with high educational attainment, of which 1.51 (95% CI: 1.45–1.57) year could be attributed to CVD. Females without schizophrenia with low educational attainment lost 2.48 (95% CI: 2.42–2.55) years compared with females without schizophrenia with high educational attainment, of which 1.03 (95% CI: 0.98–1.07) years could be attributed to CVD. Low educational attainment was not significantly associated with life years lost due to either all‐cause or CVD mortality within the schizophrenia group (Table S5). Results were similar for parental education (Table S6). For life years lost curves visualizing these results, please see Figure S2. As for the analyses including 3 categories of educational/parental education, where we compared life years lost between the highest and lowest thirds, results remained largely unchanged. For example, in the general population males and females in the lowest third of educational attainment compared with males and females in the highest third lost 4.07 (95% CI: 3.99–4.13) and 3.35 (95% CI: 3.29–3.42) years, respectively, due to all‐cause mortality, while males and females in the lowest third compared with the highest third of educational attainment in the schizophrenia group lost 1.07 (95% CI: 0.46–1.72) and −0.50 (95% CI: −1.35–0.37) years, respectively (Tables S7 and S8).

#### 3.2.3 Sensitivity analyses

When performing Cox regression and life years lost analyses with the life course SEP measure in place of education/parental education, we obtained largely similar results. For example, individuals in the low SEP group in the general population were at a higher risk of all‐cause mortality (HR = 1.46 [95% CI: 1.45–1.47]) compared with the high SEP group, whereas individuals with schizophrenia and low SEP were not at increased risk of all‐cause mortality (HR = 1.04 [95% CI: 0.94–1.15]) compared with individuals with schizophrenia and high SEP. For a more fine‐grained visualization of the associations between the continuous life course SEP scale and mortality, please see Figure S3. When adding an interaction term to the Cox model, there were significant interaction effects between schizophrenia and low SEP for all‐cause mortality (HR = 0.70 [95% CI: 0.59–0.83]) (Table S9).

In the general population males and females with low SEP compared with males and females with high SEP lost 2.23 (95% CI: 2.15–2.32) and 1.64 (95% CI: 1.56–1.72) years, respectively, due to all‐cause mortality, while males and females in the low SEP compared with the high SEP group in the schizophrenia group lost −0.86 (95% CI: −1.79–0.01) and −0.66 (95% CI: −1.75–0.53) years, respectively (Table S10).

## 4 DISCUSSION

The main aim of this study was to quantify the associations between educational attainment and mortality in schizophrenia, and to compare these associations with those found in the general population. We found that while individuals with schizophrenia had higher all‐cause and CVD mortality rates and lower educational attainment than the general population, they had a smaller association between educational attainment and mortality than the general population. While males and females with low educational attainment in the general population lost on average 3 years compared with those with high educational attainment at age 30 years, low educational attainment was not significantly associated with life years lost in those suffering from schizophrenia.

It has previously been shown that socioeconomic factors such as educational attainment, income, employment and housing conditions are associated with higher risk of schizophrenia.^13^, ^24^, ^25^ The current findings are in line with this previous research, as we found schizophrenia to be associated with lower educational attainment, as well as with lower life course SEP, which in addition to education includes household income and living conditions. (Table 1). However, to the best of our knowledge, this is the first time the impact of educational attainment has been assessed with respect to mortality in schizophrenia using individual‐level data in a nationwide population study. Another strength of the current study is the use of educational attainment in both index persons and their parents, as indicators of SEP in general are likely to be caused by reduced social functioning related to schizophrenia symptoms. For educational attainment this might be particularly relevant, as age at onset for schizophrenia has a peak around 20 years,^26^ an important period in life for attaining length of education. When we performed Cox regressions and life years lost analyses with parental educational attainment instead of educational attainment of the index persons, the results remained largely unchanged. Hence, it is unlikely that the potential reverse causation between schizophrenia and halted educational attainment results in biased estimates of the main findings in this study. As an interesting demographic finding, we observed that the educational attainment of parents of individuals with schizophrenia was similar to that of parents of individuals without schizophrenia, in contrast to the significantly lower educational attainment in individuals with schizophrenia than in those without schizophrenia. Since causation can mainly go from parental education to schizophrenia in offspring and not in the other direction, this finding, which is in line with a previous report^13^ provides additional support to the hypothesis of “social drift” in schizophrenia, as opposed to that of “social causation”, suggesting a causal directionality from schizophrenia to low educational attainment, rather than the other way around. It is also in line with studies using genetically informed methods, finding positive associations between polygenic risk for educational attainment and schizophrenia,^27^ although other studies have reported associations in the opposite direction.^28^ In order to further elucidate this issue, causal inference methods such as Mendelian randomization have been applied, shedding more light on the complex relation between educational attainment, schizophrenia and intelligence,^29^, ^30^ although without reaching a final conclusion.

In our sensitivity analyses we included a composite life course SEP measure in place of educational attainment, obtaining largely unchanged results, indicating that educational attainment is capturing a substantial and representative proportion of the socioeconomic variance relevant for this research question.

Taken together, the educational environment during upbringing as well as educational attainment throughout the life course appear to be more strongly associated with mortality in the general population than in people with schizophrenia.

There are several possible interpretations of this finding. One could speculate that the egalitarian Norwegian social security and health system reduces somatic under‐treatment and absolute poverty among individuals with schizophrenia, which could be more pronounced in countries with less socioeconomic equality. As we have quantified the impact of education‐related factors as marginal, the remaining 9–10 years of the mortality gap between individuals with schizophrenia and the general population is thus more likely related to other aspects of schizophrenia than what is encompassed by variation in educational attainment. Patient‐related factors such as an unhealthy lifestyle and system‐related factors like late recognition by healthcare providers, under‐diagnostics and under‐treatment for somatic diseases such as CVD are still plausible explanations. However, it is possible that the educational gradient for these factors is non‐existent or much smaller among individuals with schizophrenia than for the general population. In line with this assumption, individuals with schizophrenia might have reached a “ceiling effect” in terms of mediating CVD risk factors such as smoking, dyslipidemia, and obesity. Belonging to the lower end of the social strata does not necessarily add substantially to this risk due to little remaining variation, as the majority of individuals with schizophrenia are exposed to high levels of these risk factors. This interpretation is further supported by previous findings suggesting that most of the additional risk of CVD among people with low education/disadvantaged SEP in the general population is explained by increased levels of established CVD risk factors.^6^, ^16^

To gain a more complete overview on mediating factors and causal mechanisms, further studies should explore the associations between educational attainment and well‐known CVD risk factors among individuals with schizophrenia compared with the general population. It would also be highly informative to investigate whether the current findings are generalizable to other countries with less developed welfare systems, in particular as better relative outcome in schizophrenia patients have been reported from developing countries.^31^ Moreover, as premorbid cognitive dysfunction has been reported for individuals suffering from schizophrenia, it would be of great interest to include cognitive measures indexed before illness onset in the current analyses, to shed further light on the relation between schizophrenia, educational attainment and mortality.^15^ In addition to a higher load of well‐known CVD risk factors, shared genetic risk^8^ as well as adverse effects of antipsychotic medication could contribute to premature death in schizophrenia, independently of educational attainment/SEP. However, a recent Mendelian randomization study found little evidence for shared genetic risk between schizophrenia and CVD, and indicated rather that CVD results causally from the schizophrenia phenotype, with BMI and lipids as mediators.^32^ It should also be kept in mind that even if antipsychotic drugs are associated with weight gain, diabetes mellitus and dyslipidemia,^10^, ^33^ which, in turn, are established CVD risk factors, population studies have found exposure to antipsychotics to reduce the risk of all‐cause and CVD death in individuals with schizophrenia.^34^

The present study has some limitations: Firstly, the use of NIS as a source of identifying individuals with schizophrenia might have led to the misclassification of a number of patients with schizophrenia not receiving disability benefits as non‐schizophrenia individuals. As these are probably the mildest forms of schizophrenia in the high‐end part of the socioeconomic scale, our mortality estimates and associations between educational attainment and mortality might be susceptible to bias. However, our mortality rates (HRs, Table 2) and estimates of life years lost (Table S5, Figure 2) are in line with other recent studies.^2^, ^3^, ^35^, ^36^ Moreover, 94% of Norwegian schizophrenia patients have been found to be unemployed^37^ and NIS has previously been shown to be a valid data source for identification of a range of neuropsychiatric disorders,^21^, ^22^ for example, the specificity of a NIS diagnosis of cerebral palsy was 99% when compared with hospital records and parental reports.^22^ We have also previously used schizophrenia diagnoses from NIS for the purpose of investigating the relation between intrauterine growth restriction and schizophrenia.^21^ Further, when extending our analyses to the broader psychosis spectrum (F2 diagnoses from ICD‐10 and 295, 297, 298.0, 298.1 and 298.2 diagnoses from ICD‐8/9 in place of F20 and 295, respectively), the main results remained largely the same, in that we found a negative interaction between educational attainment and mortality for psychosis subjects when compared with the remaining population. Secondly, as NIS lacks data on age of onset of the disorder, we cannot include this measure in our survival models; consequently, our design included some immortal time for individuals diagnosed earlier than 1990. To estimate the potential bias inflicted by this lack of data, we have replicated a previously published Danish study^2^ with and without considering the observed age of onset, where we found that not including age of onset for schizophrenia patients resulted in an underestimation of life years lost by approximately 10%. Thus, our quantification of 9–10 years lost when comparing individuals with schizophrenia with the general population could possibly be in the range of 10–11 years. In line with this issue, as our study cohort does not include individuals below 30 years of age, this might lead to a slight underestimation of mortality estimates, although probably affecting estimates for unnatural causes more than for natural causes of death. Thirdly, as autopsies are performed in a minor fraction of deaths in Norway, there is a risk of misclassification of cause of death. Nonetheless, there is no evidence that this limitation could lead to a systematic bias for the current findings. Fourthly, we lack information on antipsychotic medication and established CVD risk factors in the current linkage, which in conjunction with genetically informed data could have enabled more detailed analyses of mediating and causal mechanisms. Fifthly, the use of a linkage of several nationwide registries imposed a challenge due to different start points and diagnostic systems. We carefully sought to balance the purposes of including information from all registries while at the same time maximizing the cohort size and statistical power, as well as minimizing the potential sources of bias.

We conclude that while individuals suffering from schizophrenia in general have lower educational attainment and higher risk of premature mortality compared with the general population, there is a smaller association between educational attainment and mortality in schizophrenia subjects than in the general population. Our findings also provide support to the social drift hypothesis in schizophrenia. Based on the current results, it is possible that social inequality including inequality in educational attainment plays a more important role with regards to mortality in the general population than in individuals with schizophrenia. Hence, one may speculate that interventions targeted at reducing social differences might be more effective in the general population than in individuals with schizophrenia, and that early interventions aiming at attenuating the illness progress per se are more important in terms of preventing CVD risk in schizophrenia, at least in a country with relative high equality in health services and education like Norway. But further exploration of these findings is needed, and may ultimately contribute to stronger evidence for policies and clinical guidelines aimed at reducing the mortality gap in schizophrenia.

## CONFLICT OF INTEREST

We declare no competing interests.

### PEER REVIEW

The peer review history for this article is available at https://publons.com/publon/10.1111/acps.13407.

## Supporting information

Appendix S1

## Data Availability

Research data cannot be shared due to ethical considerations.
