# Supplementary material for: Educational attainment and mortality in schizophrenia
Source: Acta Psychiatr Scand. 2022 Feb 18;145(5):481–93. doi: 10.1111/acps.13407 (PMC9305099; doi:10.1111/acps.13407)
Supplement: Supplementary file 1 — Appendix S1 [file ACPS-145-481-s001.docx]

**Online supplementary material**

**Educational attainment and mortality in schizophrenia,**

Tesli M, Degerud E, Plana-Ripoll O, Gustavson K, Torvik FA, Ystrom E, Ask H, Tesli N, Høye A, Stoltenberg C, Reichborn-Kjennerud T, Nesvåg R, Næss Ø

**Description:**

Table S1

Table S2

Table S3

Table S4

Table S5

Table S6

Table S7

Table S8

Table S9

Table S10

Figure S1

Figure S2

Figure S3

**Table S1**

| Sample | Predictor variable | CVD mortality (HR (95% CI) | All-cause mortality (HR (95% CI) |
| --- | --- | --- | --- |
| Total cohort (N=1852113) | Schizophrenia | 2.98 (2.80-3.18) | 2.85 (2.75-2.95) |
|  | Male sex | 2.34 (2.31-2.37) | 1.76 (1.75-1.77) |
|  | Education (0-8) | 1.21 (1.21-1.22) | 1.17 (1.17-1.18) |
|  | Schizophrenia* education | 0.85 (0.81-0.90) | 0.89 (0.87-0.92) |
| Schizophrenia patients (N=6548) | Male sex | 2.04 (1.78-2.34) | 1.62 (1.50-1.74) |
|  | Education (0-8) | 1.07 (1.02-1.13) | 1.08 (1.05-1.11) |

**Table S1.** Cox regressions mutually adjusted for schizophrenia, sex, education (likert scale from 0 to 8) and age at start for follow-up in the Norwegian population aged 30-65 Jan 1 1990 with follow-up to Dec 31 2014 (total N=1852113, N individuals with schizophrenia=6548). Left: CVD (cardiovascular disease) related mortality. Right: All-cause mortality.

**Table S2**

| Sample | Predictor variable | CVD mortality (HR (95% CI) | All-cause mortality (HR (95% CI) |
| --- | --- | --- | --- |
| Total cohort (N=1852113) | Schizophrenia | 3.37 (3.14-3.62) | 3.16 (3.03-3.28) |
|  | Male sex | 2.13 (2.10-2.16) | 1.62 (1.61-1.63) |
|  | Parents’ education (0-8) | 1.14 (1.13-1.15) | 1.09 (1.09-1.10) |
|  | Schizophrenia* parents’ education | 0.87 (0.82-0.92) | 0.91 (1.10-0.93) |
| Schizophrenia patients (N=6548) | Male sex | 2.07 (1.77-2.42) | 1.62 (1.49-1.76) |
|  | Parents’ education (0-8) | 1.04 (0.98-1.11) | 1.03 (1.0-1.06) |

**Table S2.** Cox regressions mutually adjusted for schizophrenia, sex, parents’ education (likert scale from 0 to 8) and age at start for follow-up in the Norwegian population aged 30-65 Jan 1 1990 with follow-up to Dec 31 2014 (total N=1852113, N individuals with schizophrenia=6548). Left: CVD (cardiovascular disease) related mortality. Right: All-cause mortality.

**Table S3**

| Sample | Predictor variable | CVD mortality (HR (95% CI) | All-cause mortality (HR (95% CI) |
| --- | --- | --- | --- |
| High educational attainment  N=720529 | Schizophrenia | 4.46 (3.86-5.16) | 3.86 (3.57-4.18) |
|  | Male sex | 2.79 (2.70-2.88) | 1.74 (1.72-1.77) |
| Middle educational attainment  N=521741 | Schizophrenia | 3.50 (3.12-3.93) | 3.17 (2.97-3.38) |
|  | Male sex | 2.46 (2.40-2.51) | 1.79 (1.77-1.81) |
| Low educational attainment  N=556029 | Schizophrenia | 2.49 (2.28-2.72) | 2.42 (2.31-2.56) |
|  | Male sex | 2.15 (2.12-2.19) | 1.72 (1.71-1.74) |

**Table S3.** Cox regressions mutually adjusted for schizophrenia, sex and age at start of follow-up, divided by educational attainment (high, middle and low) in the Norwegian population aged 30-65 Jan 1 1990 with follow-up to Dec 31 2014 (total N=1852113, N individuals with schizophrenia=6548). Left: CVD (cardiovascular disease) related mortality. Right: All-cause mortality.

**Table S4**

| Sample | Predictor variable | CVD mortality (HR (95% CI) | All-cause mortality (HR (95% CI) |
| --- | --- | --- | --- |
| High parental educational attainment  N=297351 | Schizophrenia | 4.33 (3.59-5.23) | 3.74 (3.39-4.12) |
|  | Male sex | 2.11 (2.03-2.19) | 1.50 (1.47-1.53) |
| Middle parental educational attainment  N=474051 | Schizophrenia | 4.13 (3.61-4.73) | 3.58 (3.32-3.85) |
|  | Male sex | 2.15 (2.10-2.21) | 1.63 (1.60-1.65) |
| Low parental educational attainment  N=731497 | Schizophrenia | 2.93 (2.67-3.22) | 2.84 (2.69-2.99) |
|  | Male sex | 2.13 (2.09-2.17) | 1.65 (1.63-1.67) |

**Table S4.** Cox regressions mutually adjusted for schizophrenia, sex and age at start of follow-up, divided by parental educational attainment (high, middle and low) in the Norwegian population aged 30-65 Jan 1 1990 with follow-up to Dec 31 2014 (total N=1852113, N individuals with schizophrenia=6548). Left: CVD (cardiovascular disease) related mortality. Right: All-cause mortality.

**Table S5**

|  |  | Life years lost to all-cause death, 95% CI) | | Life years lost to CVD death (95% CI) | |
| --- | --- | --- | --- | --- | --- |
| Sample | **Comparison** | **Males** | **Females** | **Males** | **Females** |
| Total cohort (N=1852113) | **Schizophrenia vs remaining population** | 9.96 (9.55-10.37) | 8.59 (8.08-9.10) | 3.21 (2.82-3.65) | 2.32 (1.91-2.77) |
|  | **Low education vs high education** | 3.28 (3.21-3.35) | 2.48 (2.42-2.55) | 1.51 (1.45-1.57) | 1.03 (0.98-1.07) |
| Schizophrenia (N=6548) | **Low education vs high education** | 0.53 (-0.08-1.19) | -0.41 (-1.27-0.45) | 0.37 (-0.38-1.09) | 0.34 (-0.41-1.15) |

**Table S5.** Life years lost to cardiovascular disease (CVD) and all-cause death in the Norwegian population aged 30-65 Jan 1 1990 with follow-up to Dec 31 2014 (total N=1852113, N individuals with schizophrenia=6548). CI=confidence interval.

**Table S6**

|  |  | Life years lost to all-cause death, 95% CI) | | Life years lost to CVD death (95% CI) | |  |
| --- | --- | --- | --- | --- | --- | --- |
| Sample | **Comparison** | **Males** | **Females** | **Males** | **Females** | |
| Total cohort (N=1852113) | **Schizophrenia vs remaining population** | 9⸱96 (9⸱55-10⸱37) | 8⸱59 (8⸱08-9⸱10) | 3⸱21 (2⸱82-3⸱65) | 2⸱32 (1⸱91-2⸱77) | |
|  | **Low parental education vs high parental education** | 1.77 (1.70-1.84) | 1.35 (1.28-1.43) | 0.97 (0.92-1.03) | 0.63 (0.58-0.69) | |
| Schizophrenia (N=6548) | **Low parental education vs high parental education** | 0.66 (-0.11-1.44) | -0.19 (-1.18-0.87) | -0.25 (-0.93- 0.44) | -0.47 (-1.31-0.45) | |

**Table S6.** Life years lost to cardiovascular disease (CVD) and all-cause death in the Norwegian population aged 30-65 Jan 1 1990 with follow-up to Dec 31 2014 (total N=1852113, N individuals with schizophrenia=6548). CI=confidence interval.

**Table S7**

|  |  | Life years lost to all-cause death, 95% CI) | | Life years lost to CVD death (95% CI) | |  |
| --- | --- | --- | --- | --- | --- | --- |
| Sample | **Comparison** | **Males** | **Females** | **Males** | **Females** | |
| Total cohort | **Schizophrenia vs remaining population** | 9⸱96 (9⸱55-10⸱37) | 8⸱59 (8⸱08-9⸱10) | 3⸱21 (2⸱82-3⸱65) | 2⸱32 (1⸱91-2⸱77) | |
|  | **Lowest versus highest third education** | 4.07 (3.99-4.13) | 3.35 (3.29-3.42) | 1.92 (1.86-1.98) | 1.43 (1.38-1.47) | |
| Schizophrenia | **Lowest versus highest third education** | 1.07 (0.46-1.72) | -0.50 (-1.35-0.37) | 0.92 (0.17-1.64) | 0.49 (-0.27-1.29) | |

**Table S7.** Life years lost to cardiovascular disease (CVD) and all-cause death in the Norwegian population aged 30-65 Jan 1 1990 with follow-up to Dec 31 2014. Lowest third is compared with highest third of educational attainment. CI=confidence interval.

**Table S8**

|  |  | Life years lost to all-cause death, 95% CI) | | Life years lost to CVD death (95% CI) | |  |
| --- | --- | --- | --- | --- | --- | --- |
| Sample | **Comparison** | **Males** | **Females** | **Males** | **Females** | |
| Total cohort | **Schizophrenia vs remaining population** | 9⸱96 (9⸱55-10⸱37) | 8⸱59 (8⸱08-9⸱10) | 3⸱21 (2⸱82-3⸱65) | 2⸱32 (1⸱91-2⸱77) | |
|  | **Lowest versus highest third parental education** | 2.43 (2.36-2.50) | 1.54 (1.47-1.62) | 1.43 (1.37-1.48) | 0.80 (0.74-0.85) | |
| Schizophrenia | **Lowest versus highest third parental education** | 0.09 (-0.58-0.78) | -0.26 (-1.25-0.81) | 0.56 (-0.21-1.34) | 0.37 (-0.47-1.29) | |

**Table S8.** Life years lost to cardiovascular disease (CVD) and all-cause death in the Norwegian population aged 30-65 Jan 1 1990 with follow-up to Dec 31 2014. Lowest third is compared with highest third of parents’ educational attainment. CI=confidence interval.

**Table S9**

| Sample | Predictor variable | CVD mortality (HR (95% CI) | All-cause mortality (HR (95% CI) |
| --- | --- | --- | --- |
| Total cohort (N=1852113) | Schizophrenia | 3.02 (2.78-3.28) | 2.82 (2.69- 2.95) |
|  | Male sex | 2.15 (2.12-2.18) | 1.66 (1.64-1.67) |
|  | Low SEP | 1.55 (1.52-1.57) | 1.46 (1.45-1.47) |
|  | Schizophrenia*low SEP | 0.65 (0.59-0.72) | 0.70 (0.59-0.83) |
| Schizophrenia patients (N=6548) | Male sex | 2.14 (1.76-2.56) | 1.65 (1.50-1.82) |
|  | Low SEP | 1.21 (1.02-1.43) | 1.04 (0.94-1.15) |

**Table S9.** Cox regressions mutually adjusted for schizophrenia, sex, socioeconomic position (SEP) and age at start for follow-up in the Norwegian population aged 30-65 Jan 1 1990 with follow-up to Dec 31 2014 (total N=1852113, N individuals with schizophrenia=6548). Left: CVD (cardiovascular disease) related mortality. Right: All-cause mortality. SEP = life course socioeconomic position.

**Table S10**

|  |  | Life years lost to all-cause death, 95% CI) | | Life years lost to CVD death (95% CI) | |
| --- | --- | --- | --- | --- | --- |
| Sample | **Comparison** | **Males** | **Females** | **Males** | **Females** |
| Total cohort (N=1852113) | **Schizophrenia vs remaining population** | 9.96 (9.55-10.37) | 8.59 (8.08-9.10) | 3.21 (2.82-3.65) | 2.32 (1.91-2.77) |
|  | **Low SEP vs high/middle SEP** | 2.23 (2.15-2.32) | 1.64 (1.56-1.72) | 1.01 (0.93-1.09) | 0.62 (0.56-0.67) |
| Schizophrenia (N=6548) | **Low SEP vs high/middle SEP** | -0.86 (-1.79-0.01) | -0.66 (-1.74 0.53) | 1.30 (0.25-2.22) | 0.47 (-0.45-1.46) |

**Table S10.** Life years lost to cardiovascular disease (CVD) and all-cause death in the Norwegian population aged 30-65 Jan 1 1990 with follow-up to Dec 31 2014 (total N=1852113, N individuals with schizophrenia=6548). CI=confidence interval. SEP = life course socioeconomic position.

**Figure S1**


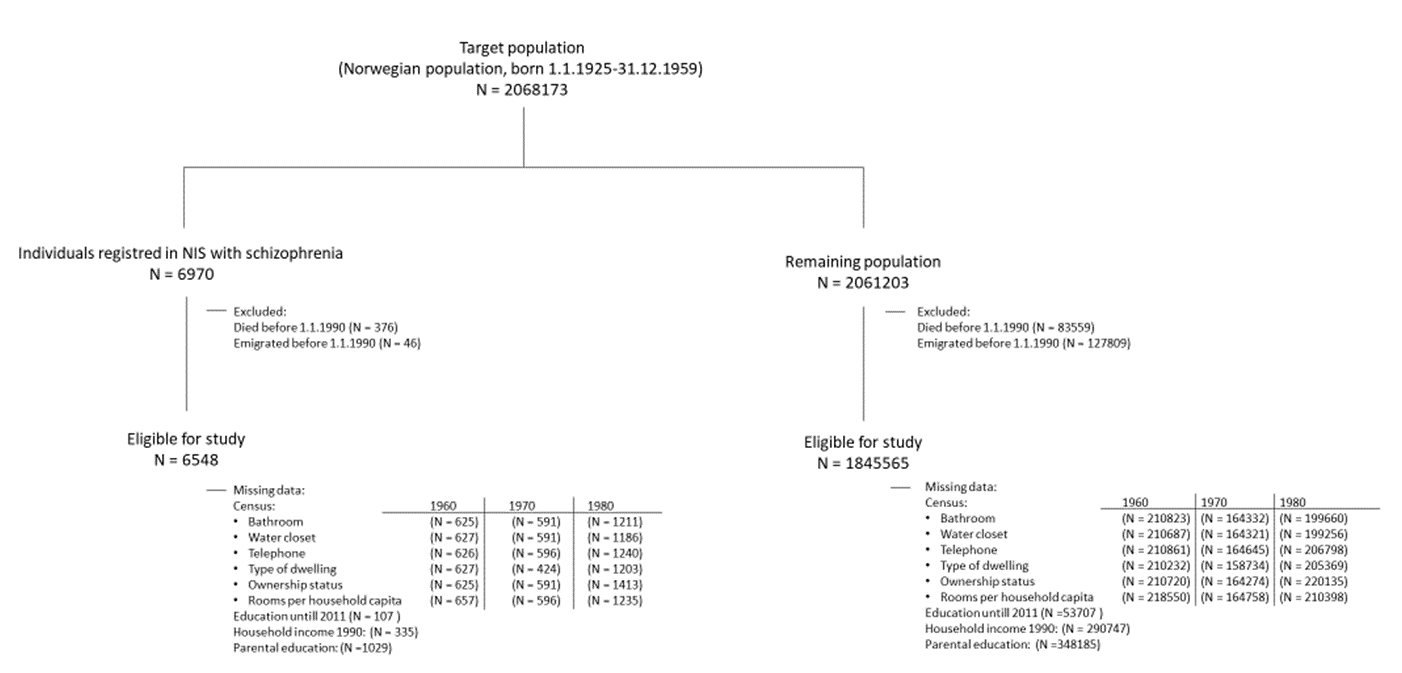


**Figure S1.** Flow chart showing inclusions and exclusions.

**Figure S2**

**
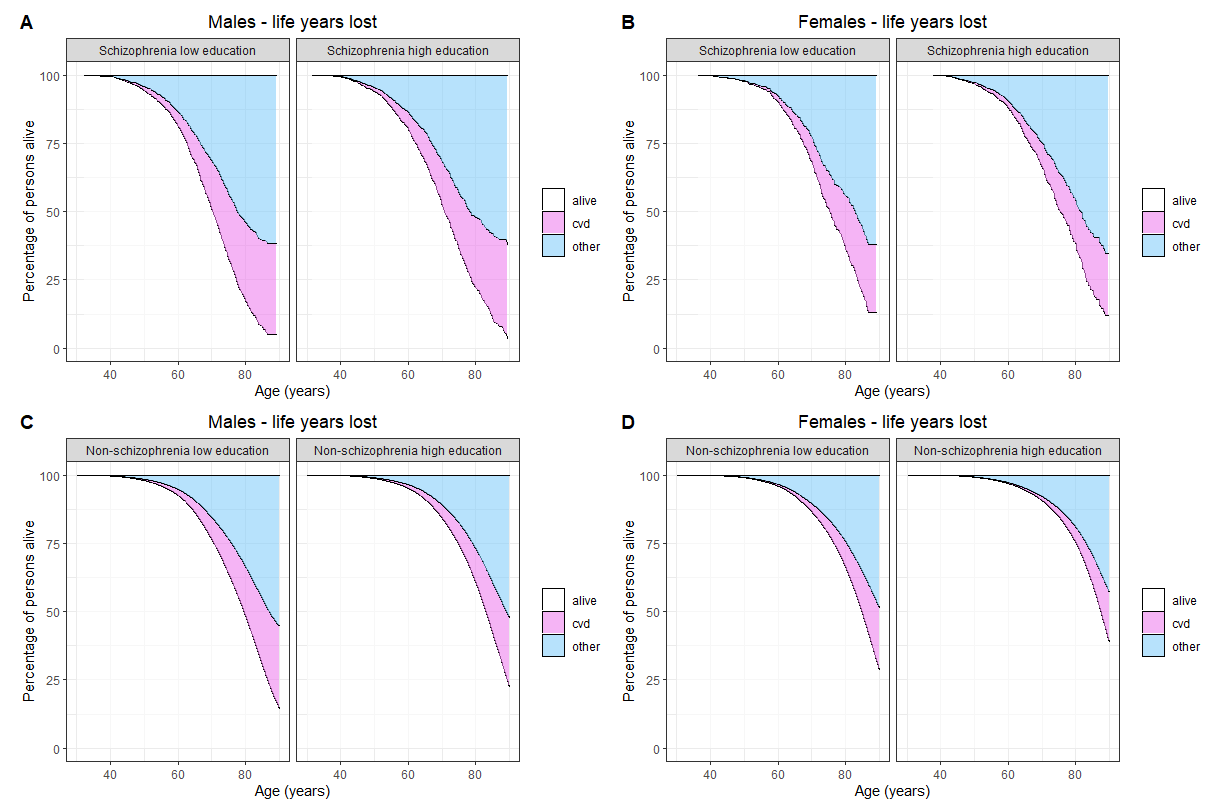
**

**Figure S2.** Excess life years lost at birth for the Norwegian population aged 30-65 Jan 1 1990 with follow-up to Dec 31 2014 (Total N=1852113, N individuals with schizophrenia=6548). Survival time x axis=years. CVD=life years lost due to cardiovascular related disease. Other= life years lost due all other causes. A: males with schizophrenia and low educational attainment compared with males with schizophrenia and high educational attainment. B: females with schizophrenia and low educational attainment compared with females with schizophrenia and high educational attainment. C: males without schizophrenia with low educational attainment compared with males without schizophrenia with high educational attainment. D: females without schizophrenia with low educational attainment compared with females without schizophrenia with high educational attainment.

**Figure S3**


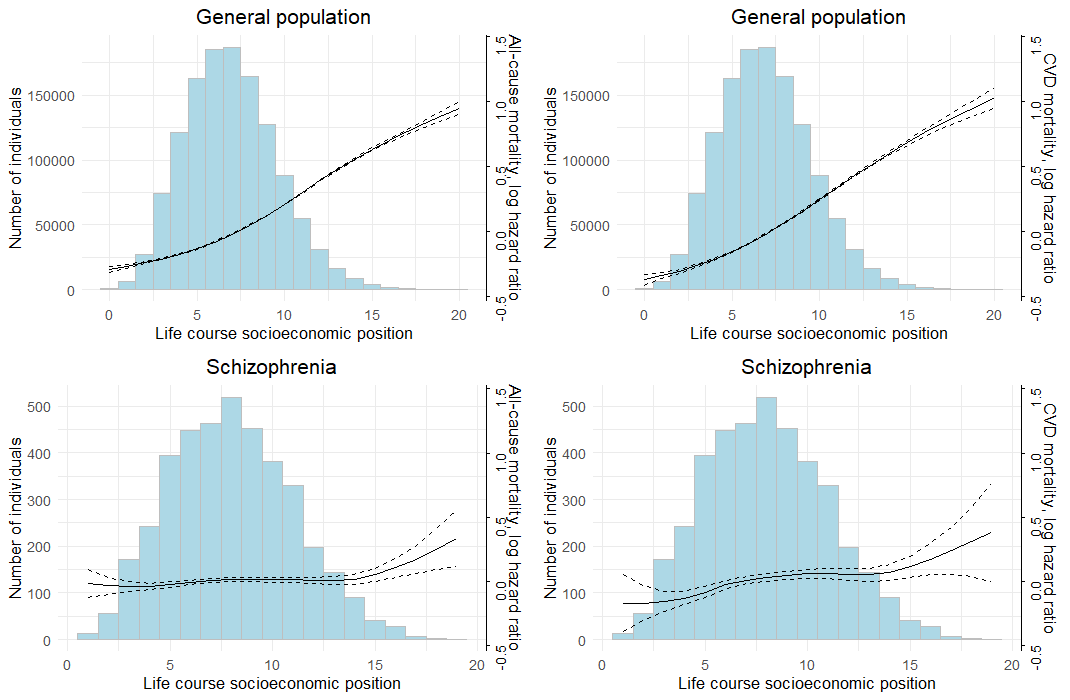


**Figure S3.** Frequency distribution of the Norwegian population (upper panel) (N=1852113), and individuals with schizophrenia (lower panel) (N=6548) born 1925-1960 with follow-up from 1990 to 2015. Index of life course socioeconomic position (SEP) (range 0±20) and (superimposed) the association of the index with the risk of all-cause (left) and cardiovascular disease (CVD) mortality (right). Cox proportional hazard model with life course socioeconomic index modelled as a penalised smoothing spline. The hazard ratio is on log scale and the relationship presented at the mean value of the covariates age and sex. High score on the life course SEP scale indicates disadvantage and low life course SEP.
